# Cluster analysis of cognitive performance in a sample of patients with Parkinson's disease

**DOI:** 10.1590/s1980-5764-2016dn1004010

**Published:** 2016

**Authors:** Carolina Pinto Souza, Guiomar Nascimento Oliveira, Maria Paula Foss, Vitor Tumas

**Affiliations:** 1 Department of Neuroscience and Behavior, Ribeirão Preto School of Medicine, University of São Paulo, Ribeirão Preto, SP, Brazil.

**Keywords:** Parkinson's disease, cognitive impairment, dementia, mild cognitive impairment, cluster analysis

## Abstract

**Background:**

Cognitive impairment is a common feature of Parkinson's disease (PD). The diagnoses of mild cognitive impairment (MCI) in patients with PD implies an increased risk for later development of dementia, however, it is unclear whether a specific type of cognitive loss confers increased risk for faster cognitive decline.

**Objective:**

Determine whether it was possible to identify distinct cognitive phenotypes in a sample of patients with PD.

**Methods:**

A cross-sectional evaluation of 100 patients with PD recruited from a movement disorders clinic was conducted. The patients were evaluated using the simplified motor score of the UPDRS, the Hoehn and Yahr, Schwab and England, Geriatric Depression Scale, Pfeffer Functional Activities Questionnaire, Clinical Dementia Rating Scale, Mini-Mental State Examination, clock drawing test, digit span, word list battery of CERAD, Frontal Assessment Battery and verbal fluency test. We classified the patients as having normal cognition (PDNC), MCI (PDMCI) or dementia (PDD). Data were analyzed using the chi-square test, non-parametric statistics and cluster analysis.

**Results:**

There were 40 patients with PDD, 39 with PDMCI and 21 with PDNC. Patients with PDD were older, had longer disease duration, lower education and lower MMSE scores. Cluster analysis showed 3 general distinct cognitive profiles that represented a continuum from mild to severe impairment of cognition, without distinguishing specific cognitive profiles.

**Conclusion:**

Cognitive impairment in PD occurs progressively and heterogeneously in most patients. It is unclear whether the definition of the initial phenotype of cognitive loss can be used to establish the cognitive prognosis of patients.

## Abstract (pt)

**Embasamento:**

O comprometimento cognitivo é um problema comum da doença de Parkinson (DP). O diagnóstico de comprometimento cognitivo leve (CCL) em pacientes com DP implica em risco aumentado para o desenvolvimento posterior de demência, no entanto, não é claro se algum tipo específico de perda cognitiva confere risco para um declínio cognitivo mais rápido.

**Objetivo:**

Determinar se seria possível identificar fenótipos cognitivos em uma amostra de pacientes com DP.

**Métodos:**

Foi uma avaliação transversal de 100 pacientes com DP recrutados de uma clínica de distúrbios de movimento. Eles foram avaliados utilizando um escore motor simplificado da UPDRS, Hoehn e Yahr, Schwab e England, Escala de Depressão Geriátrica, Questionário de Atividades Funcionais de Pfeffer, Escala CDR, Mini-Exame do Estado Mental, desenho do relógio, extensão de dígitos, lista de palavras da bateria do CERAD, bateria de avaliação frontal e teste de fluência verbal. Nós classificamos os pacientes como tendo cognição normal (PDCN), CCL (PDCCL) ou demência (PDD). Os dados foram analisados por meio do teste do qui-quadrado, estatística não-paramétrica e análise de cluster.

**Resultados:**

Havia 40 pacientes com PDD, 39 com PDCCL e 21 com PDCN. Pacientes com PDD eram mais velhos, tinham maior tempo de doença, menor escolaridade e desempenho inferior no MEEM. A análise de *cluster* mostrou 3 perfis cognitivos distintos que representariam um continuo entre discreto a grave comprometimento da cognição, sem distinguir perfis cognitivos específicos.

**Conclusão:**

O comprometimento cognitivo na DP ocorre de forma progressiva e heterogênea na maioria dos pacientes. Não é claro se a definição do fenótipo inicial de perda cognitiva poderia ser utilizado para estabelecer o prognóstico cognitivo para o paciente.

## INTRODUCTION

Cognitive impairment is a common feature of Parkinson's disease (PD). Many patients present with mild cognitive impairment (MCI) even at disease onset and most develop dementia during the course of the disease.^1,2^ Cognitive decline has a negative impact on the quality of life of patients and caregivers, increases the risk for institutionalization and death, and also significantly increases the costs of the disease.^3-5^ There are many risk factors associated with cognitive decline in PD, including age, duration of disease, severity of motor symptoms and the diagnosis of MCI.^6^

MCI in patients with PD has a cross-sectional prevalence of around 25%, and patients with PD may present impairment in many different cognitive domains such as attention, memory, visuospatial function, executive function and language.^7,8^ The clinical presentation of MCI in PD varies widely, and there is also substantial variation in the progression of cognitive deficits across patients.^2^

MCI in PD has only recently been more extensively studied. Most studies have shown that the diagnosis of MCI implies an increased risk for later development of dementia, however, it remains unclear whether a specific type of cognitive loss confers increased risk for faster cognitive decline.^2^

Observations from different studies about this issue are clearly controversial. Janvin et al. suggested that single domain non-memory MCI and multiple domains MCI were associated with later development of dementia.^9^ Hobson et al. linked impairment of memory and language to increased risk for developing dementia.^10^ Janvin et al. reported that poor performance on a test sensitive to executive dysfunction predicted later development of dementia in PD patients,^11^ while Levy et al found that impairment in verbal memory and executive function were associated with the development of dementia in patients with PD.^12^ We can conclude from these observations that it is not presently possible to define whether specific cognitive MCI profiles are associated with faster development of dementia in patients with PD.

The presentation and evolution of cognitive impairment in patients with PD appears to be heterogeneous, and it is important to determine whether MCI phenotypes can be identified that can characterize specific subgroups of patients which are more sensitive to faster conversion to dementia.

The aim of this study was to identify different cognitive profiles in a Brazilian sample of patients with PD using cluster analysis.

## METHODS

We evaluated 100 patients diagnosed with PD according to the United Kingdom Brain Bank diagnostic criteria for PD,^13^ comprising 58 males, who consecutively attended the movement disorders outpatient clinic of the Ribeirão Preto School of Medicine. Patients were evaluated by 2 neurologists (C. P. S. and G. N. O.) using many clinical tools: a simplified motor score of the UPDRS (smUPDRS), the Hoehn and Yahr and the Schwab and England scales, as well as the 15-item Geriatric Depression Scale (GDS15). The smUPDRS assessed the same signs evaluated by the Short Parkinson's Evaluation Scale but with the original 5-point items of the UPDRS.^14^ This shortened scale has shown good reliability and validity in Brazilian patients with PD.^15^ Patients presenting with *delirium*, moderate or severe hallucinations, depression, or who did not have best control of motor symptoms were not included in the study. The cognitive and functional evaluations were performed using the Pfeffer Functional Activities Questionnaire, Clinical Dementia Rating (CDR), Mini-Mental State Examination, the clock drawing test, digit span from the WAIS-III battery, word list battery of the CERAD, Frontal Assessment Battery and semantic verbal fluency test. After evaluation, patients were classified as having normal cognition (PDNC), MCI (PDMCI) or dementia (PDD) according to MDS diagnostic criteria.^16,17^ Patients with motor fluctuations were examined while in the "on state".

The data were analyzed using the Chi-square test and non-parametric Mann-Whitney test and the Kruskal-Wallis test with Dunn's *post hoc* test. Cluster analysis was used to verify the grouping of elements of the sample according to patient performance on cognitive tests. The variables for individual performance on cognitive tests were transformed into z-scores. The dissimilarity measure applied to the data was the Euclidean distance, and the technique used for the hierarchical clustering was performed using the Ward method. After establishing the number of clusters, an adjustment for the "k-means" non-hierarchical method was made. This method allows the grouping of subjects with similar characteristics, permitting changes to the individual cluster according to the homogeneity of groups. The variables of interest used to characterize patient performance on different cognitive domains were: the Mini-Mental State Examination (MMSE), Clock Drawing Test (CDT), Semantic Word Fluency (VF), Frontal Assessment Battery (FAB), Word list direct recall (directCERAD), Word list delayed recall (delayedCERAD), Word list recognition (recogCERAD), Digit span forward (DSforw), Digit span backward (DSback).

Statistical analyses were performed using the SPSS 19 software package and the level of statistical significance was defined as p<0.05. The local research ethics committee approved the study and all participants signed the informed consent form.

## RESULTS

The clinical evaluation led to the diagnosis of PDD in 40 patients (40%), PD-MCI in 39 patients (39%) and PDNC in 21 patients (21%) (Table 1). Patients with PDD were older, had longer disease duration, lower education and lower MMSE scores than those with PDMCI or PDNC. There were not significant differences among groups according to disease stage and severity of motor symptoms.

**Table 1:** Clinical and demographic data of this sample of 100 patients with PD, represented by medians (min-max).

	PDNC	PD-MCI	PDD	p
N	21	39	40	
Age (years)	54 (29-79)	61 (34-84)	69* (46-87)	<0.001*
Education (years)	5 (1-20)	5 (1-15)	3.5 (0-16)	<0.001*
Disease duration (years)	8 (3-16)	7 (2-17)	9.5* (3-19)	<0.001*
Hoehn and Yahr stage	2.0 (0-3)	2.3 (1-4)	2.0 (1-4)	0.598
UPDRS simplified motor score	12 (4-29)	12 (5-24)	11.5 (1-36)	0.537
MMSE	27 (21-30)	25 (20-30)	21* (9-29)	<0.001*

* Significant difference (p <0.05) between the PDD group compared to the PD-MCI and PDNC groups; MMSE: mini-mental state examination.

Cluster analysis was perform in order to divide the individuals of the sample into groups that were heterogeneous between one another, and which included homogeneous subjects within the same group according to individual cognitive performance. This analysis characterized three distinct groups in the sample:

– *Cluster 1:* consisting of 38 subjects: 23 patients with PDD, 13 patients with PD-MCI and 2 patients with PDNC, having a mean age of 67 years, mean education of 4 years and mean CDR of 0.6.

– *Cluster 2:* consisting of 42 subjects: 17 patients had PDNC, 25 PD-MCI, and none had PDD. This cluster comprised younger subjects (mean age=56 years), with higher education (mean=6.74 years) and lower scores on the CDR scale (mean=0.2). Based on the median and z-scores, the performance of this group showed a tendency for better results than the other groups on all cognitive tests.

– *Cluster 3:* consisting of 14 subjects: all patients with PDD. Medians and z-scores were lower on all cognitive tests than the other groups, suggesting more severe cognitive loss. The individuals in this cluster were older than those in cluster 1, and had less education (mean 3.6 years) and higher CDR than the two other groups (mean 1.5).

We found no differences in gender distribution among the three groups. There was a significant difference only in age, education and CDR (p<0.001).

**Figure 1:**
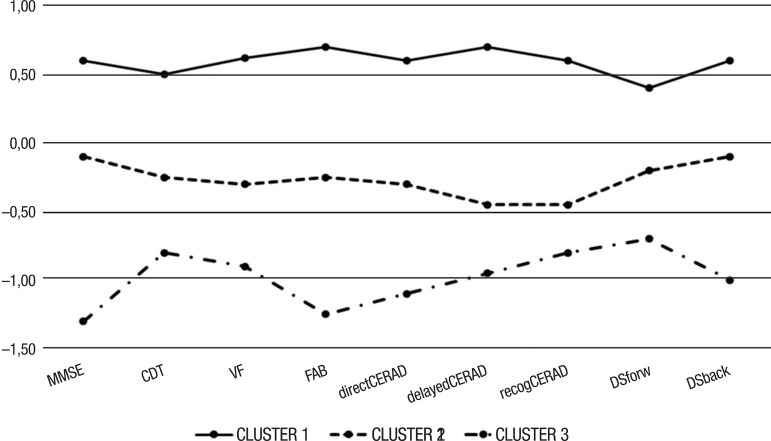
Graph illustrating z-score of each cluster in each cognitive test. The black line represents cluster 1, comprising PD-MCI patients (59%) and PDNC (41%) patients. This group had better cognitive performance than the other groups, with z-scores higher than the others on all tests. The dashed line represents cluster 3, comprising only patients with PDD and showing lower z-scores than the other groups on all tests. The dotted line represents cluster 2, comprising patients with PDD (60.5%), PD-MCI (34.2%) and PDNC (5.3%). This group had intermediate z-scores compared to the other 2 groups.

Figure 1 depicts the performance of each cluster on each cognitive test, based on its mean z-scores.

## DISCUSSION

Cognitive dysfunction is a common feature of patients with PD, as was also shown by the findings of our study. Using simple and relatively short cognitive tests, only 21 out of 100 patients were diagnosed with normal cognition. Patients diagnosed with dementia and MCI corresponded to 39% and 40% of all cases from our sample, respectively. These prevalence figures are similar to those observed in other studies evaluating patients followed in specialized centers and slightly higher than the average expected values.^8,18^ These findings were expected because a specialized clinic usually represents a sample of more complicated cases of the disease.

Many authors agree that the diagnosis of MCI implies a greater risk for developing dementia in patients with PD. However, it seems that the progression of cognitive impairment may be highly heterogeneous for only the clinical subtype of MCI. In the general population, it is clear that multiple domains MCI and greater involvement of memory are associated with an increased risk for subsequent development of dementia.^19^ In patients with PD, it is possible that factors other than the cognitive phenotype determine the risk for progression of the cognitive deficit. Cognitive deterioration in PD has several possible pathophysiological mechanisms, and it may be that the clinical presentation of cognitive deficits does not correlate directly with these. Recently, some authors have suggested that different cognitive profiles in PD could indicate distinct pathophysiological mechanisms.^20^ According to this hypothesis, executive dysfunction would reflect abnormalities of the frontostriatal projections due to striatal dopaminergic deficit while prominent memory and posterior deficits would represent Lewy body pathological deposition in cortical areas and also cortical cholinergic depletion.^20^ However, there are other observations suggesting that the pathophysiology of cognitive impairment in PD may be much more complex and unpredictable than that proposed in this dual syndrome hypothesis.^21^ Moreover, studies describing the clinical characteristics and different MCI subtypes in patients with PD have shown highly variable results.^22^ In order to understand the main determinants of onset and progression of cognitive impairment in patients with PD, larger longitudinal studies monitoring different biomarkers are required.

Another objective and simple way of analyzing this in a cross-sectional evaluation is to perform a cluster analysis based on data from cognitive tests. Some studies have used this statistical methodology to determine the existence of different clinical subtypes of PD, but this has not frequently been used to analyze the heterogeneity of the cognitive impairment in these patients. In a study of 40 patients with PD without dementia, McKinlay et al. (2009) determined the presence of three distinct cognitive profiles in this sample.^23^ These were characterized as a group of patients without cognitive impairment, a group with uncertain cognitive profile, and another group with loss in multiple cognitive domains. Dujardin et al. (2013) conducted the same analysis in a sample of 558 patients with PD.^24^ They defined 5 distinct clusters: patients in cluster 1 had no deﬁcits on any of the cognitive tests, patients in cluster 2 had lower performance than cluster 1 on some tests but still within normal limits, patients in cluster 3 had multiple domains MCI, while patients in cluster 4 and 5 had more severe cognitive impairment in multiple cognitive domains. Our findings were very similar to those observed in these two studies. Although the study of Dujardin et al. defined five different clusters,^24^ the different clusters represent general stages of progressive global cognitive deterioration and do not discriminate between specific cognitive profiles. It is presently not possible to characterize any particular pattern of cognitive impairment potentially associated with a specific cognitive prognosis. We can conclude from these observations that overall profiles of cognitive performance make up a continuum from normal cognition to dementia in patients with PD.

Another explanation for our findings is that patients with PD may be separated into different clinical subtypes, especially taking into account non-motor symptoms. Thus, our patients may have been clustered into distinct groups in relation to cognition only because they belonged to distinct clinical subtypes of PD. This way of classifying different PD subtypes is based on evidence that neuronal loss can occur differently in the different nuclei affected by the neurodegenerative process. Under these circumstances, the 3 subtypes identified by us would probably represent distinct subtypes of PD.^25^

The main limitations of our study were the small number of patients evaluated and the use of a limited battery that included only simple cognitive bedside tests.

In conclusion, cognitive impairment in PD occurs progressively and heterogeneously in most patients, and it is unclear whether the definition of the initial phenotype of cognitive loss can be used to establish the cognitive prognosis for patients.

## References

[1] Aarsland D, Bronnick K, Larsen JP, Tysnes OB, Alves G (2009). Cognitive impairment in incident, untreated Parkinson disease:
the Norwegian ParkWest study. Neurology.

[2] Goldman JG, Litvan I Mild cognitive impairment in Parkinson's disease. Minerva Med.

[3] Leroi I, McDonald K, Pantula H, Harbishettar V (2012). Cognitive impairment in Parkinson disease: impact on quality of
life, disability, and caregiver burden. J Geriatr Psychiatry Neurol.

[4] Garcia-Ptacek S, Farahmand B, Kareholt I, Religa D, Cuadrado ML, Eriksdotter M (2014). Mortality risk after dementia diagnosis by dementia type and
underlying factors: a cohort of 15,209 patients based on the Swedish
Dementia Registry. J Alzheimers Dis.

[5] Vossius C, Larsen JP, Janvin C, Aarsland D (2011). The economic impact of cognitive impairment in Parkinson's
disease. Mov Disord.

[6] Litvan I, Aarsland D, Adler CH, Goldman JG, Kulisevsky J, Mollenhauer B (2011). MDS Task Force on mild cognitive impairment in Parkinson's
disease: critical review of PD-MCI. Mov Disord.

[7] Aarsland D, Bronnick K, Fladby T (2011). Mild cognitive impairment in Parkinson's disease. Curr Neurol Neurosci Rep.

[8] Aarsland D, Bronnick K, Williams-Gray C, Weintraub D, Marder K, Kulisevsky J (2010). Mild cognitive impairment in Parkinson disease: a multicenter
pooled analysis. Neurology.

[9] Janvin CC, Larsen JP, Aarsland D, Hugdahl K (2006). Subtypes of mild cognitive impairment in Parkinson's disease:
progression to dementia. Mov Disord.

[10] Hobson P, Meara J (2004). Risk and incidence of dementia in a cohort of older subjects with
Parkinson's disease in the United Kingdom. Mov Disord.

[11] Janvin CC, Aarsland D, Larsen JP (2005). Cognitive predictors of dementia in Parkinson's disease: a
community-based, 4-year longitudinal study. J Geriatr Psychiatry Neurol.

[12] Levy G, Jacobs DM, Tang MX, Cote LJ, Louis ED, Alfaro B (2002). Memory and executive function impairment predict dementia in
Parkinson's disease. Mov Disord.

[13] Hughes AJ, Daniel SE, Kilford L, Lees AJ (1992). Accuracy of clinical diagnosis of idiopathic Parkinson's disease:
a clinico-pathological study of 100 cases. J Neurol Neurosurg Psychiatry.

[14] Rabey JM, Bass H, Bonuccelli U, Brooks D, Klotz P, Korczyn AD (1997). Evaluation of the Short Parkinson's Evaluation Scale: a new
friendly scale for the evaluation of Parkinson's disease in clinical drug
trials. Clin Neuropharmacol.

[15] Tumas V UL, Ferreira GM (2004). Utility and reliability of a simplified clinical scale for
Parkinson's disease. Arq Neuropsiquiatr.

[16] Emre M, Aarsland D, Brown R, Burn DJ, Duyckaerts C, Mizuno Y (2007). Clinical diagnostic criteria for dementia associated with
Parkinson's disease. Mov Disord.

[17] Litvan I, Goldman JG, Troster AI, Schmand BA, Weintraub D, Petersen RC (2012). Diagnostic criteria for mild cognitive impairment in Parkinson's
disease: Movement Disorder Society Task Force guidelines. Mov Disord.

[18] Aarsland D, Zaccai J, Brayne C (2005). A systematic review of prevalence studies of dementia in
Parkinson's disease. Mov Disord.

[19] Dickerson BC, Sperling RA, Hyman BT, Albert MS, Blacker D (2007). Clinical prediction of Alzheimer disease dementia across the
spectrum of mild cognitive impairment. Arch Gen Psychiatry.

[20] Kehagia AA, Barker RA, Robbins TW (2013). Cognitive impairment in Parkinson's disease: the dual syndrome
hypothesis. Neurodegener Dis.

[21] Halliday GM, Leverenz JB, Schneider JS, Adler CH (2014). The neurobiological basis of cognitive impairment in Parkinson's
disease. Mov Disord.

[22] Kalbe E, Rehberg SP, Heber I, Kronenbuerger M, Schulz JB, Storch A (2016). Subtypes of mild cognitive impairment in patients with
Parkinson's disease: evidence from the LANDSCAPE study. J Neurol Neurosurg Psychiatry.

[23] McKinlay A, Grace RC, Dalrymple-Alford JC, Roger D (2009). Cognitive characteristics associated with mild cognitive
impairment in Parkinson's disease. Dement Geriatr Cogn Disord.

[24] Dujardin K, Leentjens AF, Langlois C, Moonen AJ, Duits AA, Carette AS (2013). The spectrum of cognitive disorders in Parkinson's disease: a
data-driven approach. Mov Disord.

[25] Sauerbier A, Jenner P, Todorova A, Chaudhuri KR (2016). Non motor subtypes and Parkinson's disease. Parkinsonism Relat Disord.

